# A comparison of lithium-substituted phosphate and borate bioactive glasses for mineralised tissue repair

**DOI:** 10.1016/j.dental.2019.03.008

**Published:** 2019-06

**Authors:** Ke Zhang, Abeer Alaohali, Nuttawan Sawangboon, Paul T. Sharpe, Delia S. Brauer, Eileen Gentleman

**Affiliations:** a Centre for Craniofacial and Regenerative Biology, King’s College London, London SE1 9RT, UK; b Otto Schott Institute of Materials Research, Friedrich Schiller University Jena, Fraunhoferstr. 6, 07743 Jena, Germany

**Keywords:** Bioactive glass, Lithium, Wnt/β-catenin signalling, Regeneration

## Abstract

**Objectives:**

Wnt/β-catenin signalling plays important roles in regeneration, particularly in hard tissues such as bone and teeth, and can be regulated by small molecule antagonists of glycogen synthase kinase 3 (GSK3); however, small molecules can be difficult to deliver clinically. Lithium (Li) is also a GSK3 antagonist and can be incorporated into bioactive glasses (BG), which can be used clinically in dental and bone repair applications and tuned to quickly release their constituent ions.

**Methods:**

Here, we created phosphate (P)- and borate (B)-based BG that also contained Li (LiPBG and LiBBG) and examined their ion release kinetics and the toxicity of their dissolution ions on mouse 17IA4 dental pulp cells.

**Results:**

We found that although LiPBG and LiBBG can both quickly release Li at concentrations known to regulate Wnt/β-catenin signalling, the P and B ions they concomitantly release are highly toxic to cells. Only when relatively low concentrations of LiPBG and LiBBG were placed in cell culture medium were their dissolution products non-toxic. However, at these concentrations, LiPBG and LiBBG’s ability to regulate Wnt/β-catenin signalling was limited.

**Significance:**

These data suggest that identifying a BG composition that can both quickly deliver high concentrations of Li and is non-toxic remains a challenge.

## 1 Introdction

Wnt/β-catenin signalling is fundamental in numerous cellular processes, particularly in the context of hard tissue regeneration [1]. Indeed, regulation of Wnt/β-catenin signalling has been shown to stimulate both bone regeneration to fix metallic implants in place [2] and encourage dentine formation [3]. Wnt/β-catenin signalling can be modulated by small molecule glycogen synthase kinase (GSK3) antagonists such as BIO, CHIR99021, and Tideglusib [4]. Indeed, this strategy was employed by Neves et al. who showed that GSK3 antagonists adsorbed onto a collagen sponge and placed into a defect in a mouse molar mediated complete repair of the dentine via regulation of Wnt/β-catenin signaling [3]. As delivery in this context relied on physical adsorption to the scaffold, burst delivery of GSK3 antagonists to the injury site may have been important in stimulating dentine regeneration. However, in Neves et al.’s study, small molecule-mediated repair required a material carrier and the drug had to be delivered directly into the pulp chamber to be effective. Therefore, this strategy may be limited to a subset of clinical contexts in which the pulp chamber is already exposed. Like BIO, CHIR99021 and Tideglusib, lithium (Li) has similarly been shown to be a GSK3 antagonist, regulate Wnt/β-catenin signalling, and stimulate hard tissue formation [5,6]. Moreover, Li can potentially be incorporated into and released from existing dental materials, precluding the need for a collagen sponge to be surgically implanted into the tooth. Therefore, a material that could quickly release Li might be efficacious in regulating Wnt/β-catenin signalling and promoting tissue repair, particularly in hard tissues such as bone and teeth.

Bioactive glasses (BG) are widely used in dental and bone restorations [7,8]. BG’s bioactive properties are derived from a combination of their surface reactive properties, which allow them to directly bond to living tissue via the formation of a hydroxyapatite surface layer, and their ability to release therapeutic ions, which can direct cellular responses. Indeed, Ca, P and Si released from BG [9,10], as well as ions released from BG with various ionic substitutions, including strontium [[11], [12], [13]], fluoride [14] and cobalt [15,16], have all shown promise in regenerative applications. Most BG explored for biomedical applications, including 45S5 Bioglass^®^, are based on silicate. Li can be substituted into standard silicate-based BG in place of sodium, and we and others have shown that Li-substituted BG (LiBG) release Li ions, regulating Wnt/β-catenin signaling [17,18]. However, whilst in our hands, Li ions in the form of LiCl upregulated expression of the Wnt/β-catenin signalling target gene *AXIN2* in 17IA4 mouse dental pulp cells by as much as 15-fold, Li released from Si-based LiBG only upregulated *AXIN2* expression approximately 2-fold. This is likely because Si-based LiBG can only release a limited amount of Li because they dissolve relatively slowly and form a hydroxyapatite layer on their surface, which can hinder further release [7]. Therefore, although efficacious in many applications, when quick, high levels of ion release are required, Si-based LiBG may only be minimally efficacious. Increasing the local concentration of BG particles can yield higher levels of ion release; however, this can also produce local increases in pH. Whilst slightly alkaline conditions may be preferable for promoting the formation of biological apatites, excess alkalinity can be biologically toxic [19].

Phosphate (P) and borate (B) are alternative bases for BG and have been developed for biomedical applications [[20], [21], [22]]. Unlike Si-based BG which often dissolve slowly, phosphate (PBG) and borate (BBG) BG solubility can be tuned so that they dissolve quickly in biological solutions [[23], [24], [25], [26]], potentially allowing for quicker and higher levels of Li release. Moreover, both PBG and BBG can potentially enhance hard tissue formation themselves. P is a macronutrient essential for physiological functions such as skeletal development, mineral metabolism and cell signaling [27,28], and dietary intake of B plays important roles in bone formation and maintenance [[29], [30], [31]]. PBG have been formed with good bioactivity and biocompatibility and have been proposed for bone repair and reconstruction [32,33]. Similarly, BBG have been shown to promote the osteogenic differentiation of human mesenchymal stem cells in vitro [39], used to treat osteomyelitis in a rabbit model in vivo [34], and employed to repair calvarial defects in mice [42,43]. Moreover, BBG are potentially angiogenic [26], which when combined with LiBG, which have also been reported to be angiogenic [35], may aid in tissue regeneration. Li can easily be incorporated into melt-derived PBG and BBG (LiPBG and LiBBG), potentially combining the hard-tissue regenerating properties of PBG and BBG with the Wnt/β-catenin signalling regulatory effects of Li.

The concentration of Li that must be released from a BG to regulate the Wnt/β-catenin signalling is controversial. Patients on Li therapy for psychological disorders have concentrations of Li in their serum of ∼0.8 mM (5.5 ppm) and enhanced bone mass [36]. However, the seminal study which identified Li as regulator of Wnt/β-catenin signalling and showed Li-mediated increases in bone mass in mice, reported that 20 mM LiCl (139 ppm) was necessary to upregulate markers of osteogenesis in mouse calvarial osteoblasts [5]. When released from LiBG, Han et al. reported that just 17 ppm Li was sufficient to promote the cementogenic differentiation of periodontal ligament cells [18]. Others have similarly reported that when released from sol-gel derived BG, 5 mM Li (35 ppm) could stimulate a mouse chondrocyte cell line to form a cartilage-like matrix in pellet culture, in the absence of TGF-β3 stimulation [37]. When da Silva et al. examined the expression of *AXIN2*, which interacts with GSK3 [38] and plays a central role in Wnt/β-catenin signalling, they found that expression was only marginally upregulated at concentrations between 20 and 50 mM [17]. However, when 17IA4 cells were exposed to 100 mM LiCl (694 ppm), they upregulated expression of *AXIN2* more than 15 fold. Here, we investigated the ion release and biocompatibility of LiPBG and LiBBG to determine their potential to regulate Wnt/β-catenin signalling for hard tissue repair. We show that although LiPBG and LiBBG can quickly release high levels of Li, the concomitant high levels of P and B they release are toxic to 17IA4 mouse dental pulp cells. These findings suggest that the therapeutic potential of highly soluble LiPBG and LiBBG for regulating hard tissue regeneration via Wnt/β-catenin signalling may be limited.

## 2 Materials and methods

### 2.1 BG synthesis and characterisation

Binary glass compositions (Table 1) were chosen to maximise Li ion content in the glass and were prepared by a conventional melt-quench route. High purity precursor components H_3_BO_4_ (Riedel-de-Haen), Li_2_CO_3_ (Carl Roth) and NH_4_H_2_PO_4_ (Carl Roth) were melted in an electrical furnace at 1000–1100 °C in fused silica (PBG) or platinum (BBG) crucibles for about 1 h and subsequently quenched between brass plates to prevent crystallisation. PBG were annealed at about 400 °C and allowed to cool to room temperature overnight. Glasses were then crushed in stainless steel mortars, ground in an agate ball mill (Janetzki KM1, Germany) for 40 min and sieved to < 38 μm using stainless steel sieves. Si-based LiBG was prepared as described earlier [17] and compositions with 100% substitution of Li for Na were used here.

**Table 1:** Nominal BG compositions (in mol%) and Li content.

Glass	P_2_O_5_	B_2_O_3_	SiO_2_	Li_2_O	CaO	Li content (mg Li/mg glass)	Li content (mmol Li/mg glass)
PLi55	45			55		0.095	0.660
PLi50	50			50		0.081	0.561
BLi45		55		45		0.121	0.838
Si-LiBG	2.6		46.1	24.4	26.9	0.063	0.437

### 2.2 Cell culture

All experiments were performed with the mouse dental pulp cell line, 17IA4 [39]. Cells were cultured under standard conditions (5% CO_2_/95%, 37 °C, humidified atmosphere) in alpha minimum essential medium (αMEM) supplemented with 10% (v/v) foetal bovine serum (FBS) and 2 mM l-glutamine (all from Thermo Fisher Scientific).

### 2.3 Preparation of BG-conditioned medium

BG-conditioned media were created by soaking the indicated concentration of BG particles in αMEM. Media with BG particles were maintained on a laboratory tube roller at 37 °C for the indicated time period and then passed through a 0.22 μm syringe filter prior to ICP-MS or cell culture experiments. Measurements of pH were carried out on a standard laboratory pH metre. In some experiments, PBG was simultaneously soaked with Si-based LiBG at the indicated concentrations.

### 2.4 Elemental analysis by inductively coupled plasma mass spectrometry (ICP-MS)

Concentrations of calcium (Ca), phosphorus (P), lithium (Li), and borate (B) in cell culture media were determined by ICP-MS on a Perkin-Elmer NexION 350D using a CETAC AX520 autosampler, using customary calibration standards. Solutions were diluted 1:100 with a 1% nitric acid solution prior to analysis. Data were analysed in Syngistix software.

### 2.5 Cytotoxicity testing based on ISO10993:5

Cytotoxicity testing of dissolution ion medium was carried out as previously described [40,41], using a modified version of ISO10993:5. Positive and negative control materials were kindly provided by Raumedic AG (Helmbrechts, Germany) and consisted of organotin-stabilised polyvinyl chloride (PVC) sheet and non-toxic (Med7536 noDop) polymer tubing, respectively. To create positive and negative control media a surface area to volume ratio of 3 cm^2^/mL of control materials (sterilised in 70% ethanol for 1 h and rinsed with sterile phosphate buffered saline, PBS) were soaked in αMEM for 7 days under standard culture conditions. To test the toxicity of dissolution ion medium, 17IA4 cells were plated in 96-well plates at 20,000 cells/cm^2^ and allowed to attach for 24 h. The cell culture medium was then replaced with either positive or negative control medium or dissolution ion medium supplemented with 10% FBS and 2 mM l-glutamine. After an additional 24 h, 20 μL of a 5 mg/mL solution of MTT (3-(4,5-dimethylthiazol-2-yl)-2,5-diphenyltetrazolium bromide, Sigma) in PBS was added to each well and allowed to incubate for 4 h. Culture medium was then removed and replaced with 200 μL dimethyl sulfoxide (Sigma) and the absorbance of the product was measured on a colorimetric plate reader at 540 nm.

### 2.6 Gene expression analyses

17IA4 were plated at 25,000 cells/cm^2^ in 12 well plates in basal culture medium and allowed to attach for 24 h under standard conditions. Thereafter, the medium was replaced with conditioned or control medium and cells were cultured for an additional 24 h. 1 mL TRIzol (15596026, Thermo Fisher Scientific) was then added to each well and cell lysates stored at −20 °C. RNA was extracted according to manufacturer’s instructions, quantified by Nanodrop and reversed transcribed into cDNA using random primers (M-MLV Reverse Transcriptase Kit, Promega). qPCR was performed in a reaction mixture consisting of 5 μL SYBR Green Master Mix (KAPA SYBR**®** FAST qPCR kit), 0.1 μL forward primers, 0.1 μL reverse primers and 4.8 μL cDNA. Beta-actin (forward—GGCTGTATTCCCCTCCATCG, reverse—CCAGTTGGTAACAATGCCTGT) and *AXIN2* primers (forward—TGACTCTCCTTCCAGATCCCA, reverse—TGCCCACACTAGGCTGACA) were used. BioRad and Kappa Syber Fast (Kappa Biosystems) software were used for quantitative analysis. Reactions were performed in triplicate, and relative changes in expression of *AXIN2* to that of the beta-actin were calculated using the ΔΔC _T_ method.

### 2.7 Statistical analyses

All data are presented as means with standard deviations and were collected from at least three independent experiments. Statistical analyses were carried out using one-way analysis of variance followed by post-hoc Tukey test for multiple comparisons, or a t-test when comparing 2 groups. Differences were considered significant if p < 0.05.

## 3 Results

BG compositions are shown in Table 1; compositions with higher Li contents could not be obtained in an amorphous state owing to spontaneous crystallisation during quenching. Absolute Li content (in mg Li per mg glass) was highest in borate glass BLi45, followed by the two phosphate glasses, PLi55 and PLi50.

**Fig. 1:**
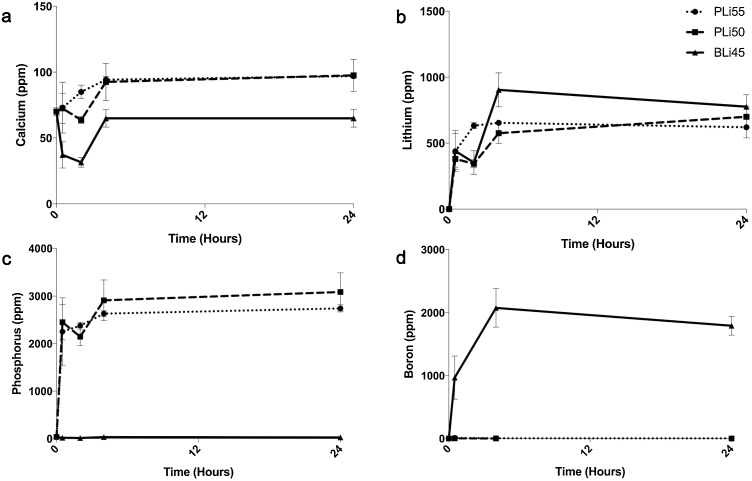
Elemental concentrations of a) Ca, b) Li, c) P and d) B in cell culture medium after 6 mg/mL LiPBG and LiBBG were soaked for up to 24 h.

We have previously shown that Li release from Si-based LiBG is both particle-size dependent and relatively slow [17]. A quicker release can be achieved by decreasing particle size, but equilibrium still requires hours (particle size <38 μm) to days (particle size 0.1–10 mm) [17]. Therefore, we first aimed to determine the Li-release profile from LiPBG and LiBBG. Both LiPBG and LiBBG dissolved quickly in cell culture medium (Fig. 1a–d). That is, within 4 h, all compositions resulted in concentrations of Li in cell culture medium of more than 500 ppm, which did not change significantly over 24 h. Similarly, concentrations of Ca, P and B in cell culture media remained stable after 4 h. These data suggest that unlike Si-based LiBG, which dissolve slowly, LiPBG and LiBBG dissolve quickly upon exposure to cell culture medium, releasing their constitutive ions. Moreover, the concentration of Li released from LiPBG and LiBBG was higher than that which has been achieved with Si-based LiBG when incubated at the same concentration (6 mg/mL). Indeed, Si-based LiBG in which 100% of the Na was substituted with Li was never able to release more than ∼300 ppm Li from the same size particles over 7 days, even when 10 times (60 mg/mL) the concentration of BG was placed in cell culture medium [17]. Therefore, LiPBG and LiBBG can achieve quicker and higher levels of Li release than Si-based LiBG.

**Fig. 2:**
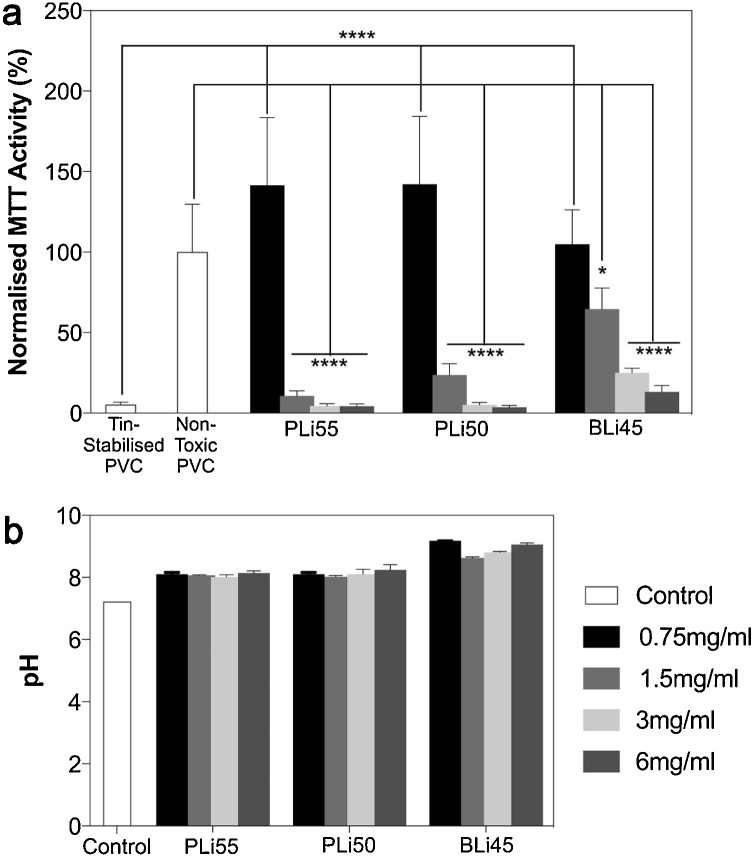
a) Normalised metabolic activity of 17IA4 cells after 24 h treatment with the dissolution products of differing concentrations of LiPBG and LiBBG. For all BG compositions at 0.75 mg/mL, metabolic activity was no different than that of negative controls. At concentrations above 0.75 mg/mL, metabolic activity was always different from that of negative controls and no different from that of positive controls, *p* <  0.05. b) pH of control (αMEM) and dissolution ion medium formed from soaking various concentrations of LiPBG and LiBBG.

We next aimed to determine whether the dissolution products from LiPBG and LiBBG were toxic. Therefore, we soaked BG particles in cell culture medium for 24 h, placed their dissolution products on 17IA4 mouse dental pulp cells and assessed their viability according to ISO10993:5. The dissolution products of 6 mg/mL LiPBG and LiBBG were highly toxic to cells. Indeed, the metabolic activity of 17IA4 cells exposed to these dissolution products were significantly different than that of negative control cells exposed to non-toxic polymers, but were no different from that of cells exposed to toxic material controls (Fig. 2a). Reducing the concentration of glass in medium to 3 mg/mL and 1.5 mg/mL could not rescue cells. Indeed, only when the concentration of LiPBG and LiBBG soaked with cell culture medium was reduced to 0.75 mg/mL did we find the metabolic activity of 17IA4 cells exposed to the dissolution products LiPBG and LiBBG to be no different from that of negative controls. Our previous work had shown that the dissolution products of Si-based LiBG were non-toxic at 6 mg/mL, but at higher concentrations, toxicity was mediated by changes in the pH of cell culture medium [17]. Therefore, to determine if changes in pH could have been responsible for the toxicity of LiPBG and LiBBG here, we tested medium pH. The pH of dissolution ion medium from all concentrations of LiPBG and LiBBG, however, did not change as a function of BG concentration (Fig. 2b), suggesting that toxicity was not mediated by changes in pH.

**Fig. 3:**
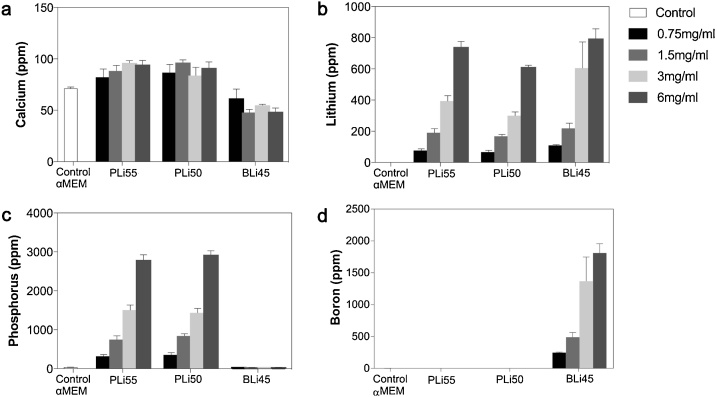
Elemental concentrations of a) Ca, b) Li, c) P, and d) B in cell culture medium after exposure to varying concentrations of LiPBG and LiBBG.

As changes in pH could not explain the toxicity of LiPBG and LiBBG, we next examined the concentrations of other ions in solution as we decreased the concentrations of LiPBG and LiBBG soaked in cell culture medium from toxic to non-toxic levels. The concentration of Ca remained stable in all groups as we decreased the concentration of BG in the medium (Fig. 3a). This is because unlike Si-based BG, LiPBG and LiBBG used here did not contain Ca and so do not release extra Ca into the medium. This was in contrast with Li concentrations, which decreased as expected for all LiPBG and LiBBG formulations (Fig. 3b). B concentrations similarly decreased as we decreased the concentration of LiBBG in media (Fig. 3d); and in LiPBG formulations, P decreased from ∼3000–4000 ppm in 6 mg/mL conditions to 300–400 ppm in 0.75 mg/mL conditions (Fig. 3c).

da Silva has previously shown that Li is non-toxic to 17IA4 cells at concentrations as high as 700 ppm [17], suggesting that toxicity could not be explained by the Li released from the BG. B is not strictly essential in mammals, but is ubiquitous in fruits, vegetables and drinking water [42] and B deficiency can negatively impact bone development and growth [29]. Nevertheless, B is also known to toxic at high concentrations [43]. Similar dose-dependent effects have also been observed with P, as whilst P is an essential nutrient and plays central roles in a myriad of biological activities, from cell metabolism to cell signalling, P is known to be toxic if present in excessive concentrations [44]. These data therefore suggest that toxicity of the LiPBG and LiBBG may have been mediated by the high concentrations of B and P released from the BG themselves.

**Fig. 4:**
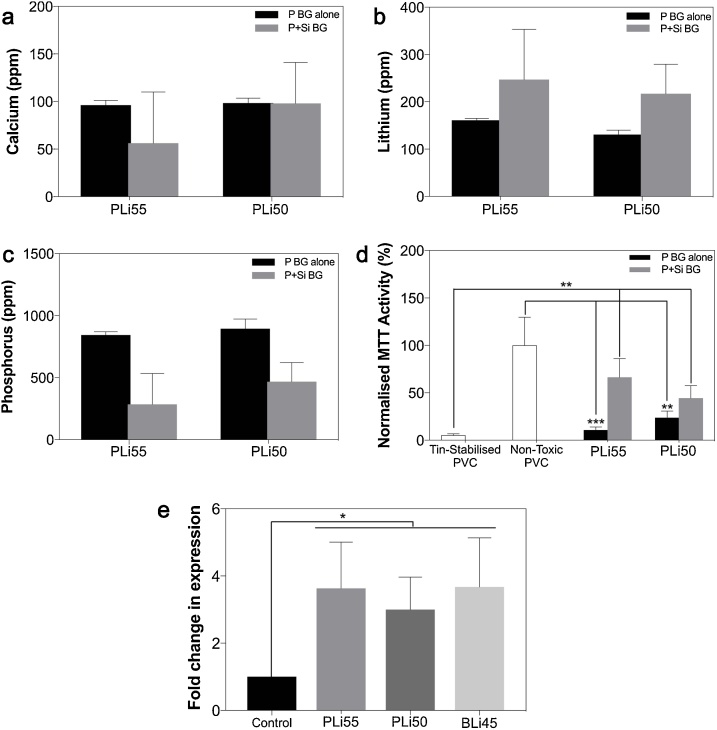
Elemental concentrations of a) Ca, b) Li and c) P in dissolution ion medium that either contained 1.5 mg/mL of LiPBG (P BG alone) or combined 1.5 mg/mL LiPBG with 4.5 mg/mL Si-based LiBG (P+Si BG). d) Normalised metabolic activity of 17IA4 cells after 24 h of treatment with the dissolution products from either medium that either contained 1.5 mg/mL of LiPBG or combined 1.5 mg/mL LiPBG with 4.5 mg/mL Si-based LiBG. Metabolic activity in both compositions of LiPBG were significantly lower than that of negative controls, even when Si-based LiBG was co-dissolved (not shown, *p* <  0.05); however, metabolic activity in the LiPBG + Si LiBG was significantly higher than that of positive (toxic) controls. e) qPCR analyses for *AXIN2* expression in 17IA4 cells treated with LiPBG and LiBBG dissolved in cell culture media at 0.75 mg/mL.

To lend further support to our hypothesis that high levels of P mediated the toxicity of their dissolution ion medium, we next combined the toxic concentration of 1.5 mg/mL LiPBG with 4.5 mg/mL Si-based LiBG to create a co-dissolution ion medium, as LiBG particles precipitate phosphorus out of cell culture medium by forming a hydroxyapatite layer on their surface as they dissolve [11]. ICP-MS analyses showed that Ca levels remained stable (Fig. 4a), and Li levels increased (Fig. 4b) when Si-based LiBG were co-dissolved with LiPBG. Moreover, in line with the expected precipitation of CaP on Si-based LiBG, the concentration of P in medium that had been conditioned with both LiPBG and Si-based LiBG decreased from ∼900 ppm to less than 500 ppm (Fig. 4c). When we repeated the ISO10993:5 toxicity test with this new co-dissolution ion medium, we found that although metabolic activity levels of cells exposed to the mixed glass medium were still significantly lower than that in negative controls, they were significantly higher than that of cells exposed to toxic positive controls (Fig. 4d). These observations strongly suggest that the toxicity of LiPBG was mediated by the high levels of P ions released from them.

To determine if non-toxic concentrations of Li released from LiPBG and LiBBG could regulate Wnt/β-catenin signalling, we next analysed *AXIN2* expression in 17IA4 cells in response to treatment with dissolution products from non-toxic 0.75 mg/mL LiPBG and LiBBG (Fig. 4e). qPCR analyses showed that *AXIN2* was upregulated approximately 3 fold in response to both LiPBG and LiBBG. These observations suggest that although only relatively low levels of Li could be released from LiPBG and LiBBG, whilst still remaining non-toxic, these levels were sufficient to mildly upregulate Wnt/β-catenin signalling.

## 4 Discussion

In order to maximise Li content in (and thus, possibly, Li ion release from) BG, binary phosphate and borate BG compositions were chosen containing Li_2_O as the only metal oxide. For LiPBG, two compositions with variable Li_2_O content (55 vs. 50 mol%) were chosen. This also potentially allows for variation of the ion release behaviour, as PBG with 45 or 50 mol% of P_2_O_5_ have been shown to differ in their dissolution behaviour [[45], [46], [47]]. The Li_2_O content in BBG and Si-based BG was lower (45 and 24.4 mol%, respectively) than in PBG, as compositions with higher contents crystallised spontaneously during preparation. Indeed, it is known that unlike for borate and silicate glasses, phosphate glasses with very high metal oxide contents can be prepared successfully [48,49].

Phosphate or borate BG differ from silicate BG in their dissolution behaviour. That is, whilst Si-based glasses release ions via an ion exchange mechanism, P- and B-based glass dissolution involves degradation of the actual glass network [45,50,51], potentially allowing for fast ion release bursts. Here, LiPBG and LiBBG released ions into cell culture medium very quickly and at higher concentrations than equivalent mass to volume ratios of Si-based LiBG. Indeed, whilst we have previously shown that 6 mg/mL of Si-based LiBG (particles 0.1–1 mm in diameter) resulted in concentrations of Li in solution of ∼5 ppm [17], placing the equivalent concentrations of LiPBG and LiBBG (particles <38 μm in diameter) in cell culture medium yielded between ∼600 and 800 ppm Li. However, whereas the dissolution products from as much as 60 mg/mL of Si-based LiBG was non-toxic to cells, the dissolution products of just 6 mg/mL LiBBG and LiPBG were highly toxic. Indeed, the toxicity of dissolution products from LiPBG and LiBBG were no different from negative controls only when we used a fraction of the concentration that for Si-based LiBG remained non-toxic. Therefore, whilst 6 mg/mL of LiPBG and LiBBG can release levels of Li that we have previously shown to strongly regulate Wnt/β-catenin signalling [17], such dissolution products are highly toxic. Only when dissolution products from far lower concentrations of LiPBG and LiBBG particles were employed, were they non-toxic. However, the levels of Li released under these conditions (although still higher than those released from Si-based LiBG) were still relatively low. Taken together, these observations suggest that we have not identified an ideal Li-containing BG composition that can both quickly release high concentrations of Li and remain non-toxic to cells.

Others have reported on the development and characterisation of PBG [21,22] and BBG [20,26,52,53] for biomedical use. In line with our observations, Salih et al. reported that PBG with high dissolution rates were toxic to 2 human osteoblast-like cell lines [22]. However, in studies in which BBG have been formed into scaffolds and cements, authors report little to no cell toxicity. These observations suggest that the solubility of the BG and the context within which it is employed are key in PBG and BBG toxicity.

Our elemental analyses of LiPBG and LiBBG dissolution ion medium combined with ISO10993:5 tests using medium in which LiPBG and Si-based LiBG were co-dissolved, suggest that LiPBG and LiBBG toxicity was mediated by the high levels of P and B released from the BG. The concentration of B in ground water varies from <0.3 to >100 mg/L, but is generally in the range of 1 mg/L [54], and its concentration in soil varies between 2 and 100 ppm [55]. This results in serum levels in healthy individuals ranging between approximately 30 and 200 μg/L [56]. There are no recommended intake levels for boron, but adults generally consume 1–2 mg/day [57], and upper limits are suggested to be no more than 20 mg/day [58]. The concentrations of B in dissolution ion media that were toxic to cells in our experiments were orders of magnitude higher than that which would be expected in serum due to normal exposure. When we lowered the concentration of LiBBG in cell culture medium; however, we were able to identify a level at which dissolution products were no longer toxic. Indeed, at 3 mg/mL (1368 ppm), media were highly toxic. When 1.5 mg/mL of LiBBG were added (490 ppm), toxicity was much abated; however, at 0.75 mg/mL (247 ppm) dissolution products were no longer toxic. The concentration of B in these dissolution products and our observations of their toxicity were directly in line with the predicted fatal serum concentrations of B in humans, which have been reported to be 50 mg/dL [59] (500 ppm). Similarly, whilst P is an essential nutrient which normally exists in human serum in the range of 2.5–4.5 mg/dL; it can also be toxic at high concentrations. Although the pathophysiology of phosphorus imbalances in the body are complex, the concentrations released from LiPBG that we report here to be toxic to 17IA4 cells are far higher than physiological levels, which are normally tightly controlled [60].

When we co-dissolved LiPBG with Si-based LiBG we were able to achieve higher levels of Li release than LiPBG compositions alone and reduced levels of toxicity, likely owing to precipitation of toxic P out of solution. These observations suggest that although a single optimised composition of BG that can both quickly release high levels of Li and remain non-toxic to cells remains elusive, combinations of Si-based LiBG, LiPBG and LiBBG may counterbalance some of the negative aspects of some compositions whilst harnessing their positive effects. Future strategies may find success pursuing such combinations.

Despite the fact that LiPBG and LiBBG that released high levels of Li also released toxic levels of B and P, dissolution ion medium created from lower concentrations of LiPBG and LiBBG were still able to upregulate *AXIN2* expression in 17IA4. Therefore, although we were unable to create a BG that both quickly released large amounts of Li without releasing toxic levels of P and B, LiPBG and LiBBG may still find use in regulating hard tissue formation via modulating Wnt/β-catenin signalling. This is in keeping with previous reports that Li-releasing BG can upregulate Wnt/β-catenin [17] and stimulate tissue regeneration [61,62], even when Li levels are relatively low.

## 5 Conclusions

Here we developed BG based on B and P that could quickly release high levels of Li, which is a known GSK3 antagonist, and thus regulate Wnt/β-catenin signalling. Our findings show that whilst it was possible to quickly release high levels of Li from both LiPBG and LiBBG, they concomitantly released toxic levels of P and B, limiting their utility in hard tissue repair. We conclude that although it was possible to create non-toxic LiPBG and LiBBG that could subtly upregulate *AXIN2* expression, we have not yet identified an ideal BG formulation that can both provide a burst release of high levels of Li whilst not simultaneously releasing toxic levels of B and P.
